# Outcomes for women with BMI>35kg/m^2^ admitted for labour care to alongside midwifery units in the UK: A national prospective cohort study using the UK Midwifery Study System (UKMidSS)

**DOI:** 10.1371/journal.pone.0208041

**Published:** 2018-12-04

**Authors:** Rachel Rowe, Marian Knight, Jennifer J. Kurinczuk, on behalf of the UK Midwifery Study System (UKMidSS)

**Affiliations:** National Perinatal Epidemiology Unit, Nuffield Department of Population Health, University of Oxford, Oxford, United Kingdom; Case Western Reserve University, UNITED STATES

## Abstract

**Objective:**

To describe and compare outcomes in severely obese (body mass index (BMI)>35kg/m^2^) women and other women admitted to alongside (co-located) midwifery units (AMU) in the United Kingdom.

**Methods:**

We carried out a national prospective cohort study using the UK Midwifery Study System (UKMidSS) in all 122 AMUs in the UK. We identified and collected data about 1122 severely obese women admitted to an AMU, 1^st^ January-31^st^ December 2016, and 1949 comparison women (BMI≤35kg/m^2^), matched on time of admission, and used Poisson regression to calculate relative risks adjusted for maternal characteristics.

**Results:**

92% of the severely obese cohort had BMI 35.1-40kg/m^2^. Severely obese multiparous women were no more likely than comparison women to experience the composite primary outcome (one or more of: augmentation, instrumental birth, Caesarean, maternal blood transfusion, 3rd/4th degree tear, maternal admission to higher level care) (5.6% vs. 8.1%, aRR = 0.68, 95% CI 0.44–1.07). For severely obese nulliparous women we found a non-significant 14% increased risk of the primary outcome (37.6% vs 34.8%, aRR = 1.14, 95% CI 0.97–1.33). High proportions of severely obese women had a ‘straightforward vaginal birth’ (nulliparous 67.9%; multiparous 96.3%). Severely obese women were more likely than comparison women to have an intrapartum Casearean section, but Caesarean section rates were low and the absolute difference small (4.7% vs 4.1%; aRR = 1.62; 95% CI 1.02–2.57). In nulliparous women, severely obese women were more likely to have an urgent Caesarean section (12.2% vs. 6.5%, aRR = 1.80, 95% CI 1.05–3.08), or a PPH≥1500ml (5.1% vs. 1.7%, aRR = 3.01, 95% CI 1.24–7.31).

**Conclusions:**

We found no evidence of significantly increased risk associated with planning birth in an AMU for carefully selected multiparous severely obese women, with BMI 35.1-40kg/m^2^. Severely obese nulliparous women have a potential increased risk of having a more urgent Caesarean section or severe PPH compared with other women admitted to AMUs.

## Introduction

The prevalence of obesity in pregnant women is increasing across the world, representing an increasing clinical and economic burden on health services.[1, 2] In the UK in 2015–16, less than half of pregnant women had a body mass index (BMI) within the normal range (18.5-25kg/m^2^) and around 8% of women giving birth had a BMI≥35 at booking.[3] Maternal obesity is a recognised risk factor for a range of complications and adverse outcomes of pregnancy, labour and birth.[4, 5] Some dietary and lifestyle interventions in pregnancy are effective in reducing maternal weight gain, but there is only weak evidence about their effectiveness in improving outcomes for women and their babies.[5–7] It is therefore increasingly important to identify how best to provide care and improve outcomes for obese pregnant women.

UK national clinical guidelines recommend that women with a BMI>35 kg/m^2^ at booking should plan birth in a consultant-led obstetric unit (OU), rather than in a midwifery-led setting, in order to reduce the associated risks.[8] There is some evidence, however, that selected sub-groups of obese women may have lower intrapartum-related risks than previously thought.[9–11] It has been suggested that some women with BMI>35kg/m^2^ might be considered suitable for planning birth in a midwifery unit (MU), as one approach to improving outcomes.[9] In healthy women with straightforward pregnancies, planned birth in a MU is associated with a more positive experience and a reduced risk of medical intervention, with no difference in safety compared with planned OU birth.[8, 12] In the UK, midwifery units may be ‘freestanding’ (FMU), located on a site geographically separate from an OU, or ‘alongside’ (AMU), on the same site or in the same hospital as an OU.[13] In both types of MU, care is provided by midwives and transfer to an OU is required for obstetric, medical or anaesthetic care (including regional analgesia).[8] Since 2008–10 the number of women giving birth in MUs in the UK has increased; in 2015 14% of all births in England took place in MUs, with around 85% of those births in AMUs.[14]

In this study we aimed to explore and describe clinical characteristics, and maternal and perinatal outcomes, in women with a BMI>35kg/m^2^ at booking admitted for labour care to an AMU in the UK and to compare outcomes in this group with women with a booking BMI≤35kg/m^2^ admitted for labour care to the same AMUs.

## Methods

### Ethics

The UK Midwifery Study System (UKMidSS) and the UKMidSS Severe Obesity Study received ethics approval from the National Research Ethics Service (NRES) Committee South West–Frenchay (REC ref. 15/SW/0166) in May 2015.

### Study design

We carried out a national prospective cohort study, identifying and collecting data about all women with a booking BMI>35kg/m^2^ admitted for labour care in all AMUs across the UK between 1^st^ January and 31^st^ December 2016, and a comparison cohort (BMI≤35kg/m^2^), matched on time of admission to the same AMUs.

### Data collection

This study was carried out using UKMidSS, a research infrastructure covering all 122 AMUs in the UK in 2016 (101 in England, 10 in Wales, 6 in Scotland and 5 in Northern Ireland). Eligible units which opened or closed during the study period participated while they were open.

UKMidSS methods have been described elsewhere.[15] In each AMU one or more midwives were UKMidSS ‘reporters’ for that unit and, in response to monthly emails from the UKMidSS co-ordinating centre at the National Perinatal Epidemiology Unit, reported the number of women with booking BMI>35kg/m^2^ (referred to in this paper as ‘severely obese’ women) admitted for labour care to the AMU in the previous month (including zero if they had no severely obese women to report). They also reported ‘denominator data’ on total admissions and births in the AMU each month. On reporting a severely obese woman, electronic case report forms (CRFs) were automatically generated in a secure web-based environment to collect further detailed anonymous information confirming the eligibility of the woman, socio-demographic and clinical characteristics, pregnancy and labour care, and maternal and neonatal outcomes. Reporters were also asked to identify and enter data on two comparison women for each severely obese woman, selected as the two women, with booking BMI≤35kg/m^2^, who were admitted to the AMU immediately before the severely obese woman. All data were anonymous and entered directly from women’s notes and/or hospital electronic patient records.

### Outcomes

The primary outcome was a composite measure of adverse maternal outcome, reflecting the need for obstetric care (i.e. that the woman could not continue to be cared for in an AMU), comprising at least one of the following: augmentation with syntocinon, instrumental delivery, intrapartum Caesarean section, general anaesthesia, maternal blood transfusion, third/fourth degree perineal tear, maternal admission for higher level care (high dependency/intensive care) in the immediate postnatal period. The use of this primary outcome, which was defined a priori before the start of data collection,[15] also facilitated comparison with Hollowell et al (2013) which investigated outcomes for ‘otherwise healthy’ obese women planning birth in OUs.[9]

We also investigated a number of secondary outcomes. While most of these are described as ‘maternal’ outcomes, several have potential implications for neonatal wellbeing, e.g. instrumental delivery, Caesarean section, shoulder dystocia. The maternal outcomes investigated were: the individual components of the composite outcome; transfer from the AMU to the care of an obstetrician during labour or within 24 hours of birth; documented shoulder dystocia; immersion in water during labour; birth in water; ‘straightforward vaginal birth’ (i.e. birth without forceps, ventouse or Caesarean, with no third/fourth degree perineal tear and no blood transfusion);[16] urgent Caesarean section (i.e. ‘category 1: immediate threat to the life of the woman or fetus’, or ‘category 2: maternal or fetal compromise which is not immediately life-threatening’;[17]) postpartum haemorrhage (PPH) ≥1500ml; maternal death.

The neonatal outcomes investigated were: Apgar score <7 at 5 minutes; initiation of breastfeeding; neonatal unit admission; stillbirth/neonatal death.

In addition to ‘negative’ outcomes indicating potential adverse events during labour and birth we explicitly chose to investigate some ‘positive’ outcomes, e.g. straightforward vaginal birth, immersion in water during labour, birth in water and initiation of breastfeeding, because of their potential impact on women’s labour and birth experience.[18]

### Data and definitions

Booking BMI>35kg/m^2^ was chosen as the criteria for inclusion in this study because UK national clinical guidance on planning place of birth recommends that women with a BMI>35 kg/m^2^ at booking should be advised to plan birth in an OU, rather than in a midwifery-led setting.[8] In the UK, BMI is determined from weight measured at ‘booking’, which is the first antenatal appointment with a midwife, at or around 8–12 weeks’ gestation. Broadly speaking BMI>35kg/m^2^ equates to obese class II (35–39.99kg/m^2^) and class III (≥40kg/m^2^) in the WHO BMI classification.[19] Women with BMI = 35kg/m^2^, considered by WHO to be obese class II, were included in our comparison group because according to national guidance they would be considered eligible for birth in a midwifery unit.

We asked UKMidSS reporters to report all women with a booking BMI>35kg/m^2^ who were admitted for labour care to an AMU and who went on to give birth in the same admission, irrespective of where they gave birth. Women admitted for assessment who went home before giving birth or who were seen only for obstetric triage were therefore not included.

All data were entered into electronic CRFs by UKMidSS reporters from women’s medical records. For a woman reported as having a BMI>35kg/m^2^ we queried any missing BMI data or data entered as ≤35, on the basis that this woman would not be eligible for inclusion in the severely obese cohort, or entered as >45, on the basis that these values were potentially implausible. Where BMI was entered as ≤35 and confirmed as correct by the reporting midwife, or missing and confirmed unavailable, the woman was excluded as ‘ineligible’. Where BMI was entered as >45, but the data query was not responded to, the BMI was regarded as ‘unconfirmed’ and the woman’s data were not included in the dataset. For a woman in the comparison group, if BMI data was missing from the electronic CRF the woman’s data were included in the dataset if the reporting midwife was able to confirm that the woman’s BMI was not >35, e.g. by checking with the midwife who looked after the woman in labour.

UK national guidelines recommend that women with specified medical or obstetric risk factors (including BMI>35kg/m^2^) identified prior to the start of labour are advised that planning birth in an OU would be expected to reduce their risk of adverse outcome.[8] However, local guidelines and individual care plans give scope for selected women with pre-existing risk factors to plan birth in midwifery-led settings; in 2008–10 around 4% of women admitted to AMUs in England for labour care had pre-existing risk factors.[16, 20] We therefore collected data about any medical or obstetric risk factors known prior to the start of labour care, in addition to raised BMI for the severely obese women, which were entered into the CRF as free text. These were independently coded by a research midwife and RR with reference to the risk factors listed in UK national guidelines indicating increased risk suggesting planned birth in an OU (Tables 39 and 40 [8]). Where the free text provided clear evidence of a medical or obstetric risk factor, e.g. “Asthma requiring hospitalisation during pregnancy”, this was coded as a ‘clear’ risk factor. Where there was insufficient information to confirm the risk factor, e.g. “Asthma”, this was coded as a ‘possible’ risk factor. Any discrepancies in coding were resolved by discussion.

We also collected data about ‘complicating conditions’ identified at the start of care in labour, using a list of complications, from the same UK national guideline, which indicate that transfer to obstetric-led care should be considered.[8] These are distinct from pre-existing risk factors identified during pregnancy and include, for example, maternal tachycardia or pyrexia, hypertension, proteinuria, prolonged rupture of membranes, abnormal presentation and fetal heart rate abnormalities.

The woman’s occupation (or her partner’s where the woman was out of work or where her occupation was not known) was used to derive the three-class version of the National Statistics Socio-economic Classification (NS-SEC), using the ‘simplified method’.[21] To derive a measure of area deprivation UKMidSS reporters entered women’s postcodes into a bespoke ‘look-up’ website which returned a ‘score’ for the Children in Low-income Families Local Measure, which they then entered into the CRF with other data. This score represents the proportion of children aged under 16 living in households in receipt of out of work benefits, or in receipt of tax credits where their reported income is less than 60% of UK median income.[22] Cut-offs derived using data on the number of babies in 2015 in the UK from official birth records were used to create deciles and quintiles.

Measurement of estimated blood loss (EBL) is likely to have been based on clinical estimation. We collected data on PPH≥1500ml, rather than EBL, because of well documented difficulties in estimating blood loss volume accurately, and evidence that estimations become more reliable as blood loss volume increases.[23] This enabled us to identify and quantify the risk of major haemorrhage, likely to require treatment to prevent serious morbidity.

### Statistical analysis

We used Stata 13SE for all analyses. We estimated the prevalence of severe obesity in women admitted for labour care in AMUs using the total reported number of women admitted for labour care to AMUs as the denominator and the total number of confirmed severely obese women as the numerator, with 95% confidence intervals (CI).

We described the maternal socio-demographic and clinical characteristics and maternal and neonatal outcomes of the severely obese and comparison cohorts by parity using frequencies and percentages.

We used log Poisson regression to calculate the relative risk (RR) of the primary and secondary outcomes in the severely obese cohort relative to the comparison group adjusted (aRR) for maternal age, ethnic group, area deprivation quintile (Children in Low-Income Families Local Measure), gestation at admission, the presence of pre-existing medical and obstetric ‘risk factors’ in addition to raised BMI, and parity where appropriate (see S1 Table for categorisation). All analyses were carried out separately by parity and in the overall group combining nulliparous and multiparous women (adjusted for parity). We used the Wald test to investigate interaction between obesity group and parity and where no interaction was identified we report pooled estimates in addition to estimates by parity. We used robust variance estimation to allow for the clustering of women within units.

During the course of the study we became aware that in some centres comparison women had not been selected according to instructions. We contacted all centres and asked reporters to confirm the selection method used for comparison women for each severely obese woman. Where comparison women were selected using an unacceptable method (14 centres, 208 comparison women), or where the selection method was unknown (10 centres, 63 comparison women), the data for these comparison women were removed from the dataset. For the primary outcome, we carried out a sensitivity analysis restricting the study population to those severely obese women for whom comparison women were selected using the specified method.

We conducted a pre-specified sub-group analysis repeating the main analysis for the primary outcome in the sub-group of ‘otherwise healthy’ severely obese women, that is, severely obese women identified as not having any pre-existing risk factors in addition to BMI>35kg/m^2^ and women in the comparison cohort with no pre-existing risk factors.

Finally, we carried out a series of *post hoc* analyses using the Chi-squared test. We tabulated reasons for Caesarean section and reasons for transfer in severely obese women and the comparison group; explored associations between reasons for transfer, obstetric interventions and mode of birth and severe PPH in severely obese nulliparous women; and tabulated place of birth in severely obese nulliparous women who experienced a severe PPH.

For all analyses using the primary outcome we used p<0.05 to assess statistical significance and, because of multiple testing, for all secondary outcomes we used p<0.01; absolute p-values are reported throughout.

### Sample size and power

Within a one-year period we anticipated approximately 600 severely obese women would be admitted to AMUs for labour care, based on estimated incidence of 1% in a population of an estimated 60,000 women admitted to AMUs.[9] For an outcome with an incidence of 20% in the comparison group this would give 80% power at the 5% level of significance to detect a RR of 1.3 or greater in the severely obese group. The actual number of severely obese and comparison group women identified during the study period generated 80% power at the 5% level to detect a RR of 1.2 or greater in the severely obese group. For a less common outcome, with an incidence of 3% in the comparison group, the study had 80% power at the 5% level to detect a RR of 1.7 or greater.

### Patient and public involvement (PPI)

PPI representatives advised on the development of the funding application for this study. The UKMidSS Steering Group includes two lay members who have represented the views of pregnant women and families throughout the conduct and interpretation of this study. We are also working with a wider PPI group to inform the development of user-friendly materials which will be used to disseminate the study results directly to women.

## Results

### Response and prevalence of severe obesity

All 122 AMUs in the UK participated in the study (100% of eligible units), with 99% response to monthly report requests.

**Fig 1:**
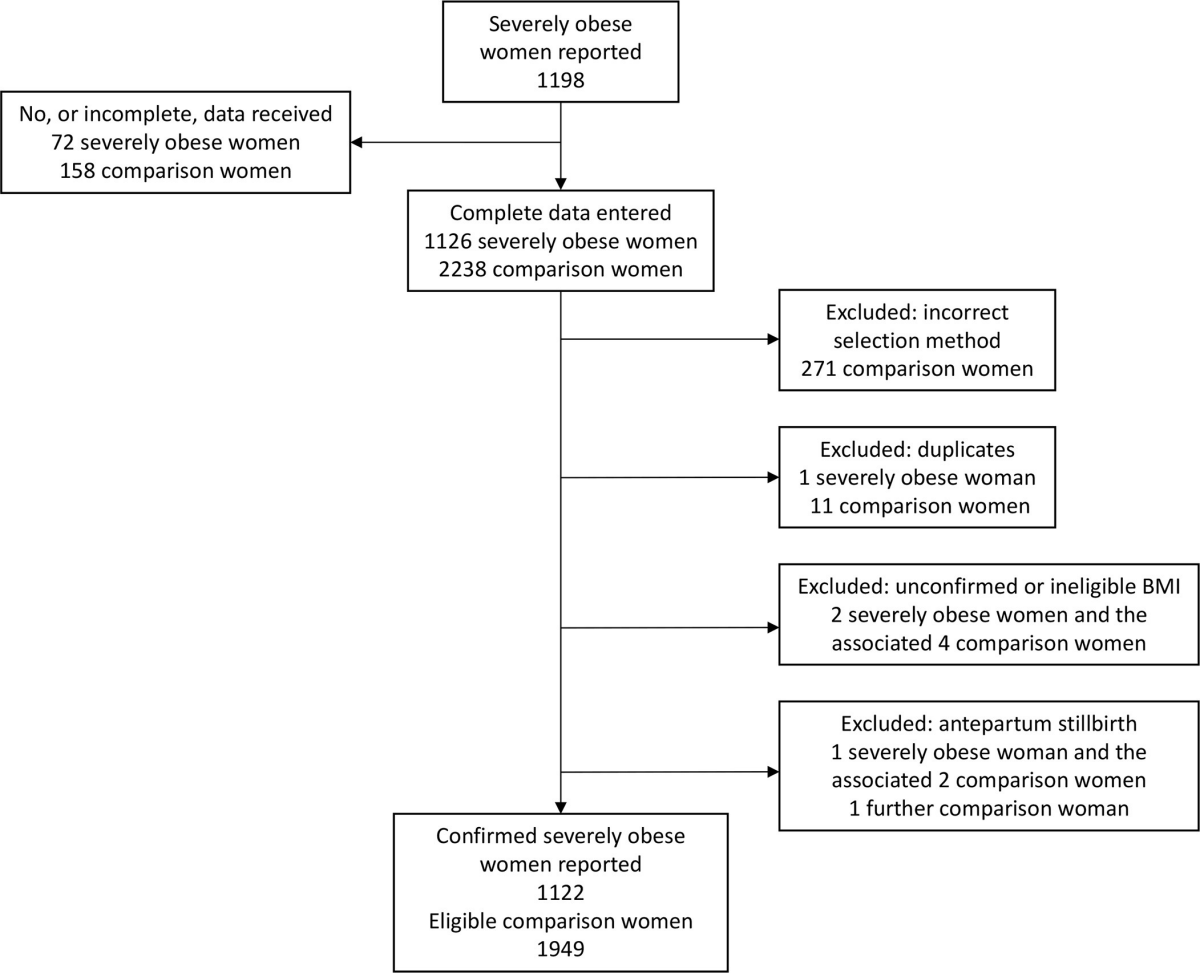
Flow diagram.

A total of 1198 severely obese women were reported (Fig 1). We received complete data for 1126 severely obese women and 2238 comparison women. After exclusions there were 1122 confirmed severely obese women for whom we had complete data, from a total of 126,524 women admitted over the year, giving an estimated national prevalence of 0.89% (95% CI 0.84–0.94). Overall 91 AMUs (75%) admitted at least one confirmed severely obese woman for labour care in 2016. The prevalence of ‘severe obesity’ in women admitted to each AMU ranged from 0–5.2%, with a median of 0.58% (IQR 0.02%, 1.15%).

### Maternal characteristics

**Fig 2:**
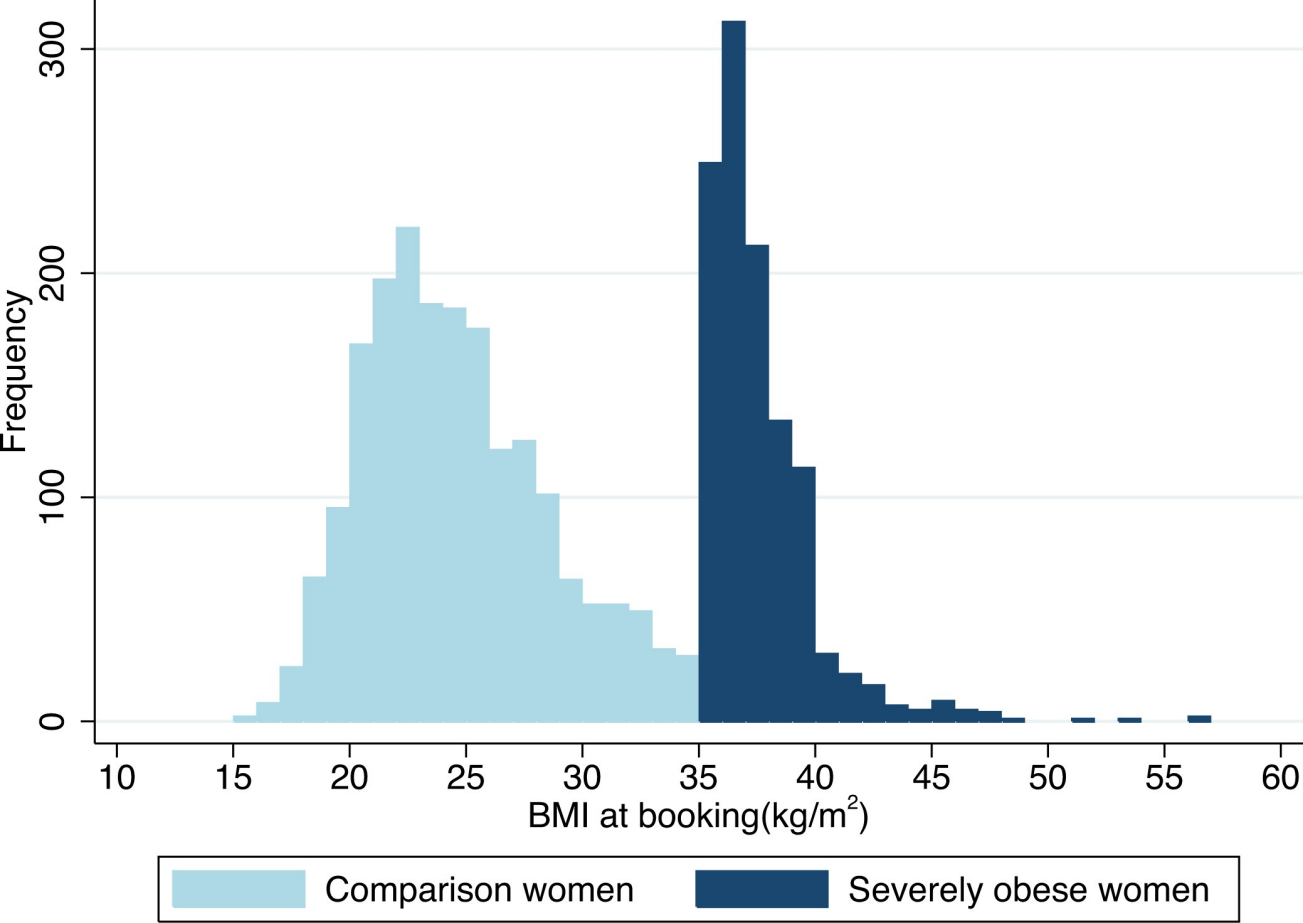
Body mass index (BMI) distribution in severely obese and comparison women. Compared with the comparison women, the severely obese women were more likely to be multiparous, have a gestational age of 41–42 weeks, and to live in a deprived area (Table 1). In multiparous women only, the severely obese women were younger and of lower socio-economic status than the comparison women. Both nulliparous and multiparous severely obese women were more likely to have at least one pre-existing medical or pregnancy risk factor (in addition to raised BMI) and to have a ‘complicating condition’ identified at the start of labour care (Table 2). A higher proportion of the severely obese women (2.1%) were identified as having gestational diabetes, compared with the comparison group (0.4%).

In the severely obese women, BMI ranged from 35.1–56.7 with a median of 37kg/m^2^, compared with 24 kg/m^2^ (range 15.5–35) in the comparison group (Fig 2). Only 88 (8%) of the severely obese women had a BMI>40 kg/m^2^.

**Table 1:** Characteristics of severely obese and comparison women, and their babies, by parity.

	Severely obese women						Comparison group					
	Nulliparous n = 312		Multiparous n = 808		All n = 1122		Nulliparous n = 890		Multiparous n = 1056		All n = 1949	
	n	%	n	%	n	%	n	%	n	%	n	%
**BMI at booking (kg/m**^**2**^**)**												
<18.5					0		44	5.0	35	3.3	79	4.1
18.5–24.9					0		505	56.9	562	53.2	1069	54.9
25–29.9					0		261	29.4	323	30.6	585	30.0
30–35.0					0		78	8.8	136	12.9	214	11.0
35.1–40.0	289	92.6	743	92.0	1034	92.2					0	
40.1–45.0	20	6.4	53	6.6	73	6.5					0	
>45.0	3	1.0	12	1.5	15	1.3					0	
Missing	0		0		0		2		0		2	
**Maternal age (years)**												
Under 20	20	6.4	7	0.9	27	2.4	76	8.5	12	1.1	88	4.5
20–24	79	25.3	125	15.5	204	18.2	189	21.2	126	11.9	315	16.2
25–29	119	38.1	279	34.5	399	35.6	299	33.6	320	30.3	620	31.8
30–34	72	23.1	260	32.2	333	29.7	240	27.0	365	34.6	607	31.1
35–39	21	6.7	123	15.2	144	12.8	79	8.9	215	20.4	294	15.1
40+	1	0.3	14	1.7	15	1.3	7	0.8	18	1.7	25	1.3
Missing	0		0		0		0		0		0	
**Ethnic group**												
White	268	85.9	680	84.2	950	84.7	738	82.9	792	75.0	1533	78.7
Asian	12	3.9	58	7.2	70	6.2	77	8.7	168	15.9	245	12.6
Black	9	2.9	39	4.8	48	4.3	20	2.3	31	2.9	51	2.6
Other	15	4.8	25	3.1	40	3.6	36	4.0	53	5.0	89	4.6
Not recorded	8	2.6	6	0.7	14	1.3	19	2.1	12	1.1	31	1.6
Missing	0		0		0		0		0		0	
**Socioeconomic status**												
Higher managerial	87	34.7	140	22.8	227	26.2	260	39.6	245	30.6	506	34.7
Intermediate	71	28.3	151	24.6	222	25.7	151	23.0	217	27.1	368	25.2
Routine and manual	75	29.9	249	40.6	325	37.6	202	30.8	282	35.3	486	33.3
Unemployed/student	18	7.2	73	11.9	91	10.5	43	6.6	56	7.0	99	6.8
Missing	61		195		257		234		256		490	
**Area deprivation quintile**^a^												
1^st^ (least deprived)	56	18.3	128	16.0	184	16.6	247	28.3	257	24.7	505	26.3
2^nd^	66	21.6	136	17.0	202	18.2	170	19.5	210	20.2	380	19.8
3^rd^	58	19.0	155	19.3	213	19.2	164	18.8	173	16.6	338	17.6
4^th^	66	21.6	184	22.9	251	22.6	139	15.9	201	19.3	340	17.7
5^th^ (most deprived)	60	19.6	199	24.8	259	23.4	152	17.4	201	19.3	354	18.5
Missing	6		6		13		18		14		32	
**Smoking status**												
Non-smoker during pregnancy	265	84.9	634	78.5	900	80.2	732	82.3	854	81.0	1589	81.6
Smoker during pregnancy	44	14.1	160	19.8	205	18.3	137	15.4	180	17.1	317	16.3
Not recorded	3	1.0	14	1.7	17	1.5	21	2.4	21	2.0	42	2.2
Missing	0		0		0		0		1		1	
**Previous pregnancies >24 weeks**												
0	312	100.0	0		312	27.9	890	100.0	0		890	45.7
1	0		469	58.0	469	41.9	0		687	65.1	687	35.3
2	0		213	26.4	213	19.0	0		244	23.1	244	12.5
3 or more	0		126	15.6	126	11.3	0		125	11.8	125	6.4
Missing					2						3	
**Gestation at admission (weeks)**												
36–37	14	4.5	34	4.2	48	4.3	46	5.2	45	4.3	91	4.7
38	40	12.9	76	9.4	116	10.4	68	7.7	113	10.7	181	9.3
39	61	19.6	182	22.6	243	21.7	233	26.3	271	25.8	505	26.0
40	115	37.0	300	37.2	416	37.2	330	37.3	416	39.5	747	38.5
41–42	81	26.1	214	26.6	296	26.5	209	23.6	207	19.7	416	21.4
Missing	1		2		3		4		4		9	
**Birthweight (g)**												
<2500	1	0.3	3	0.4	4	0.4	8	0.9	4	0.4	12	0.6
2500–2999	37	11.9	65	8.1	102	9.1	128	14.5	114	10.8	242	12.5
3000–3499	117	37.5	249	30.9	366	32.7	400	45.2	402	38.1	804	41.4
3500–3999	114	36.5	331	41.1	446	39.8	284	32.1	372	35.3	657	33.8
4000–4499	35	11.2	132	16.4	168	15.0	57	6.4	141	13.4	198	10.2
≥4500	8	2.6	26	3.2	34	3.0	8	0.9	21	2.0	29	1.5
Missing			2		2		5		2		7	

^a^ Area deprivation quintiles created using the Children in Low-Income Families Local Measure[22]

**Table 2:** Clinical ‘risk’ characteristics in severely obese and comparison women by parity.

	Severely obese women						Comparison group					
	Nulliparous n = 312		Multiparous n = 808		All n = 1122		Nulliparous n = 890		Multiparous n = 1056		All n = 1949	
	n	%	n	%	n	%	n	%	n	%	n	%
**Pre-existing risk factors**^a^												
None	288	92.3	690	85.5	980	87.4	851	95.6	950	90.1	1803	92.7
One or more clear risk factor	14	4.5	57	7.1	71	6.3	18	2.0	42	4.0	60	3.1
One or more possible risk factor	10	3.2	60	7.4	70	6.2	21	2.4	62	5.9	83	4.3
Missing	0		1		1		0		2		3	
**Gestational diabetes**												
Had oral glucose tolerance test	248	79.7	673	83.3	922	82.3	162	18.2	276	26.2	439	22.6
Gestational diabetes identified	3	1.0	21	2.6	24	2.1	4	0.5	3	0.3	7	0.4
**‘Complicating conditions’ identified at start of labour care**^b^												
None	247	79.4	725	89.7	974	86.9	780	87.6	979	92.7	1762	90.4
One or more	64	20.6	83	10.3	147	13.1	110	12.4	77	7.3	187	9.6
Missing	1				1		0		0		0	

^a^ Classified as ‘clear’ risk factor where text indicated one or more medical condition or obstetric factor indicating increased risk suggesting planned birth in an obstetric unit in national guidelines.[8] Classified as ‘possible’ risk factor where text suggests risk factor, but information insufficient to confirm clear risk factor.

^b^ Presence of any of the complications indicating need for transfer to obstetric-led care in national guidelines [8]

### Maternal interventions and outcomes

#### Primary outcome

In nulliparous women 37.6% of the severely obese group experienced our composite primary outcome, comprising at least one of augmentation, instrumental birth, Caesarean, maternal blood transfusion, 3rd/4th degree tear and maternal admission to higher level care, compared with 34.8% of the comparison group (aRR = 1.14; 95% CI 0.97–1.33) (Table 3). This difference was not statistically significant, but our analysis was almost certainly underpowered to detect a difference of this magnitude in the nulliparous group as statistically significant. Severely obese multiparous women were no more likely than comparison women to experience the primary outcome (5.6% vs. 8.1%; aRR = 0.68; 95% CI 0.44–1.07); the absolute risks of the primary outcome in multiparous women were substantially lower compared with nulliparous women (Table 3).

**Table 3:** Adverse maternal outcome in severely obese women and comparison women, by parity.

	Events	Births			Unadjusted		Adjusted^a^	
	n	n	%	(95% CI)	RR	(95% CI)	RR	(95% CI)
**Adverse maternal outcome composite**^b^								
Wald test for interaction								p = 0.03^c^
Nulliparous								
Comparison group	309	889	34.8	(31.6–37.9)	1		1	
Severely obese women	117	311	37.6	(32.2–43.0)	1.08	(0.94–1.25)	1.14	(0.97–1.33)
Multiparous								
Comparison group	85	1053	8.1	(6.4–9.7)	1		1	
Severely obese women	45	806	5.6	(4.0–7.2)	0.69	(0.45–1.06)	0.68	(0.44–1.07)

^a^ Adjusted for maternal age, ethnic group, Children in Low Income Families Measure quintile, gestation at admission, risk status and parity where appropriate

^b^ Comprising: augmentation, instrumental birth, Caesarean, maternal blood transfusion, 3^rd^/4^th^ degree tear, maternal admission to higher level care

^c^ *p* value for interaction, adjusted for maternal age, ethnic group, Children in Low Income Families Measure quintile, gestation at admission, risk status and parity (binary)

Our results were not materially different in the sensitivity analysis restricting the severely obese group only to those severely obese women for whom comparison women were selected using an appropriate method (S2 Table) and in our pre-specified sub-group analysis repeating the main analysis in ‘otherwise healthy’ women, that is women not identified as having any pre-existing risk factors (in addition to raised BMI for the severely obese group) (S3 Table).

#### Secondary maternal outcomes

We found no statistically significant differences between the two groups for the following secondary maternal outcomes: transfer to obstetric care, shoulder dystocia, augmentation with syntocinon, general anaesthesia, vaginal birth, straightforward vaginal birth, instrumental birth, 3^rd^/4^th^ degree perineal trauma, maternal blood transfusion, maternal admission for higher level care (S4 Table). High proportions of severely obese women had a ‘straightforward vaginal birth’ without instrumental assistance, with no third/fourth degree perineal trauma and no blood transfusion (nulliparous 67.9%; multiparous 96.3%) (S4 Table).

Severely obese women were significantly less likely than the comparison group to use immersion in water during labour (aRR = 0.76; 95% CI 0.64–0.90) and to give birth in water (aRR = 0.70; 95% CI 0.55–0.89) (Table 4). Nevertheless, relatively high proportions of severely obese women used immersion in water during labour (nulliparous 32.4%; multiparous 24.0%) or for birth (nulliparous 13.2%; multiparous 15.4%).

**Table 4:** Selected secondary maternal outcomes in severely obese and comparison women.

	Events	Births			Unadjusted		Adjusted^a^	
	n	n	%	(95% CI)	RR	(99% CI)	RR	(99% CI)
**Immersion in water during labour**								
Overall								
Comparison group	738	1938	38.1	(35.9–40.2)	1		1	
Severely obese women	289	1107	26.1	(23.5–28.7)	0.69	(0.57–0.82)	0.74	(0.62–0.89)
Wald test for interaction								p = 0.38^b^
Nulliparous								
Comparison group	421	883	47.7	(44.4–51.0)	1		1	
Severely obese women	99	309	32.0	(26.8–37.3)	0.67	(0.49–0.91)	0.70	(0.53–0.93)
Multiparous								
Comparison group	316	1052	30.0	(27.3–32.8)	1		1	
Severely obese women	189	796	23.7	(20.8–26.7)	0.79	(0.63–0.98)	0.80	(0.64–1.00)
**Birth in water**								
Overall								
Comparison group	407	1949	20.9	(19.1–22.7)	1		1	
Severely obese women	166	1121	14.8	(12.7–16.9)	0.71	(0.55–0.91)	0.70	(0.55–0.89)
Wald test for interaction								p = 0.96^b^
Nulliparous								
Comparison group	177	890	19.9	(17.3–22.5)	1		1	
Severely obese women	41	311	13.2	(9.4–17.0)	0.66	(0.41–1.07)	0.68	(0.43–1.07)
Multiparous								
Comparison group	229	1056	21.7	(19.2–24.2)	1		1	
Severely obese women	124	808	15.4	(12.9–17.8)	0.71	(0.54–0.92)	0.72	(0.56–0.93)
**Intrapartum Caesarean section**								
Overall								
Comparison group	80	1949	4.1	(3.2–5.0)	1		1	
Severely obese women	53	1122	4.7	(3.5–6.0)	1.15	(0.72–1.84)	1.62	(1.02–2.57)
Wald test for interaction								p = 0.86^b^
Nulliparous								
Comparison group	73	890	8.2	(6.4–10.0)	1		1	
Severely obese women	43	312	13.8	(9.9–17.6)	1.68	(1.06–2.67)	1.62	(0.98–2.67)
Multiparous								
Comparison group	7	1056	0.7	(0.17–1.2)	1		1	
Severely obese women	10	808	1.2	(0.47–2.0)	1.88	(0.56–6.21)	1.80^c^	(0.52–6.24)
**Category 1 or 2 Caesarean section**								
Overall								
Comparison group	63	1949	3.2	(2.4–4.0)	1		1	
Severely obese women	46	1122	4.1	(2.9–5.3)	1.27	(0.79–2.03)	1.79	(1.10–2.93)
Wald test for interaction								p = 0.86^b^
Nulliparous								
Comparison group	58	890	6.5	(4.9–8.1)	1		1	
Severely obese women	38	312	12.2	(8.5–15.8)	1.87	(1.15–3.03)	1.80	(1.05–3.08)
Multiparous								
Comparison group	5	1056	0.5	(0.06–0.9)	1		1	
Severely obese women	8	808	1.0	(0.3–1.7)	2.09	(0.52–8.36)	2.10^c^	(0.48–9.11)
**Postpartum haemorrhage ≥1500ml**								
Wald test for interaction								p = 0.002^b^
Nulliparous								
Comparison group	15	890	1.7	(0.8–2.5)	1		1	
Severely obese women	16	312	5.1	(2.7–7.6)	3.04	(1.34–6.92)	3.01	(1.24–7.31)
Multiparous								
Comparison group	21	1055	2.0	(1.1–2.8)	1		1	
Severely obese women	15	806	1.9	(0.9–2.8)	0.93	(0.41–2.13)	0.89	(0.41–1.94)

^a^ Adjusted for maternal age, ethnic group, Children in Low Income Families Measure quintile, gestation at admission, risk status, and parity where appropriate

^b^ *p* value for interaction, adjusted for maternal age, ethnic group, Children in Low Income Families Measure quintile, gestation at admission, risk status and parity (binary)

^c^ Adjusted for maternal age, ethnic group, gestation at admission, risk status, and parity only because of small numbers

Severely obese women were significantly more likely than the comparison group to have an intrapartum Caesarean section, although the absolute difference in the overall (combined parity) group was small (4.7% vs. 4.1%; aRR = 1.62; 95% CI 1.02–2.57) (Table 4). In nulliparous women only, the severely obese group was 80% more likely than the comparison group to have an urgent (category 1 or 2) Caesarean section (12.2% vs. 6.5%; aRR = 1.80; 95% CI 1.05–3.08) and their risk of having a PPH ≥1500ml was 3 times higher (5.1% vs. 1.7%; aRR = 3.01; 95% CI 1.24–7.31) (Table 4).

Our exploratory *post hoc* analyses showed that there were some differences in the reasons for transfer (S5 Table). Compared with the comparison group, slightly higher proportions of severely obese women were transferred because of concerns about maternal (18% vs 12.2%) or fetal (30.0 vs24.7%) wellbeing during labour, and lower proportions were transferred for delay in labour (24.0% vs 28.6%) and epidural/pain relief (11.2% vs 15.6%). There were no statistically significant differences in the reasons for Caesarean section between the two groups (S5 Table).

In the nulliparous severely obese group, all but three of the sixteen women who had a PPH≥1500ml gave birth in the OU, having been transferred from the AMU during labour (S6 Table). Eight of these women were transferred because of delay in labour (50%), compared with 11.5% in the nulliparous severely obese women who did not have a PPH≥1500ml (p<0.001). High proportions of those who had a PPH≥1500ml had their labour augmented (56.3% vs 18.6% in those who did not have a PPH≥1500ml; p<0.001) or had an epidural (81.3% vs 27.5%; p<0.001), and only 31.3% had a spontaneous vaginal birth (compared with 74.7%; p = 0.001) (S6 Table).

There were no maternal deaths in either group.

### Neonatal outcomes

We found no statistically significant associations between severe obesity and any of the neonatal outcomes studied: Apgar<7 at 5 minutes, initiation of breastfeeding and neonatal unit admission (S7 Table). There were no intrapartum stillbirths in either group, and the one neonatal death was as a result of a congenital anomaly, and was not related to antenatal/intrapartum care.

## Discussion

### Main findings

Despite national guidance,[8] the admission of women with BMI>35kg/m^2^ to AMUs in the UK is widespread, with around 75% of units admitting at least one severely obese woman for labour care during 2016. Over 90% of these women had a BMI between 35.1-40kg/m^2^, so they are not representative of the general population of severely obese pregnant women.[3]

In multiparous women we found that the severely obese group were no more likely than other multiparous women admitted to AMUs to experience an obstetric intervention or adverse maternal outcome, after adjustment for maternal characteristics, and almost all (96%) had a ‘straightforward vaginal birth’. We found no evidence of increased risk in any adverse maternal or perinatal outcomes, compared with other multiparous women admitted to AMUs.

In nulliparous women we found that the severely obese group had a non-significant 14% increased risk of experiencing an obstetric intervention or adverse maternal outcome, but our analysis was underpowered to detect a difference of this magnitude as statistically significant. Severely obese nulliparous women also had an 80% higher risk of having an urgent Caesarean section and a three-fold increased risk of having a PPH ≥1500ml, compared with other nulliparous women. For other secondary maternal and neonatal outcomes we found no associations with severe obesity in nulliparous women.

Overall, we found that severely obese women were more likely than the comparison group to have an intrapartum Caesarean section, but overall rates of Caesarean were low and absolute differences small. Severely obese women were less likely than other women to use immersion in water during labour or to give birth in water, but overall proportions of severely obese women using water for labour or birth were relatively high (26% and 15% respectively).

### Strengths and limitations

The major strength of this study is its national population-based design, which reduces the risk of the biases associated with local, hospital-based studies. All eligible UK AMUs participated in the study, with a 99% response to monthly report requests and complete data returned for over 90% of the severely obese women reported, reducing the possibility of selection bias. We were dependent on data that were routinely recorded in women’s notes so did not have data on a number of factors of interest including, for example, whether the severely obese woman was admitted to the AMU in accordance with AMU admission guidance or under a specific care plan. Although we collected data on minimum and maximum weight, only 23% of women were weighed more than once during pregnancy so we were unable to investigate associations between weight gain during pregnancy and outcomes.

By engaging with UKMidSS reporters, using reminders and checking with those units who reported no severely obese women during the study, we aimed to identify all severely obese women admitted for labour care to AMUS. Some severely obese women may have been missed and we had no other sources of data against which to validate reported cases, but our prevalence estimate is similar to that derived from data from the *Birthplace* study in 2008–10.[20]

Our aim was to compare outcomes for severely obese women admitted for labour care in AMUs with other women admitted to AMUs. We cannot therefore compare outcomes for severely obese women directly with outcomes for similar women admitted to OUs. Nevertheless, by using the same primary outcome we can, for example, compare with findings from Hollowell et al[9] and our study provides more information to help women make informed decisions about place of birth.

Because 92% of the severely obese women admitted to AMUs had a BMI between 35.1-40kg/m^2^, and were otherwise ‘selected’ for admission to an AMU in ways that we could not measure, the findings of our study should not be considered generalisable to women with BMI>40kg/m^2^. Our study was underpowered for the primary outcome in the nulliparous group, in large part because the primary outcome was less common than we anticipated, and for uncommon adverse outcomes, so findings in relation to these should also be treated with caution.

### Other evidence and clinical implications

Our findings are consistent with other evidence which suggests that selected groups of ‘lower risk’ obese and severely obese women may have lower intrapartum-related risks than previously thought.[9–11] In our severely obese cohort admitted to AMUs we observed lower rates of obstetric intervention and adverse maternal outcome overall compared with the ‘otherwise healthy’ women with BMI>35kg/m^2^ planning OU birth in the study by Hollowell et al[9] (37.7% vs 57.1% respectively in nulliparous women and 5.4% vs 21.0% respectively in multiparous women). We also observed substantially lower rates of augmentation (11.5% vs 27.2% respectively), instrumental delivery (4.9% vs 9.4%) and intrapartum caesarean section (4.7% vs 13.6%) in our severely obese cohort compared with the women in the study by Hollowell et al.[9] This is consistent with ‘lower risk’ severely obese women being ‘selected’ for AMU birth, for which we have some supporting evidence given that 92% of the severely obese cohort had a BMI between 35.1-40kg/m^2^. However, over 10% of the severely obese cohort also had one or more potential risk factor in addition to raised BMI, including 2% with gestational diabetes. It is also possible that having been admitted to the AMU, the severely obese women in our study received more attention from their carers or more individualised care, precisely because they were severely obese, but we lack the data to confirm or refute this.

Nulliparous obese women are more likely to have slow labour or ineffective uterine contractility, compared with multiparous obese women,[24, 25] with an increased risk of intrapartum Caesarean section as a consequence, which is what we observed in this study.[25, 26] Our study was underpowered for our primary outcome in nulliparous women so we are unable to say with confidence whether the nulliparous severely obese group had an increased risk of this pre-specified composite of ‘obstetric intervention or adverse maternal outcome’. We did however find that severely obese nulliparous women in the cohort were at an increased risk of severe PPH. While this was not entirely explained by their increased risk of intrapartum Caesarean section, our post hoc exploratory analyses in this small group of women are indicative of poorer uterine contractility/uterine atony, leading to delay in labour, with consequent transfer, augmentation of labour and a high chance of instrumental or operative birth. It may be reassuring that 80% of the severely obese nulliparous women who experienced a severe PPH gave birth in an OU, following transfer during labour from the AMU. Considering absolute risks in this context may also be helpful. The proportion of severely obese nulliparous women experiencing an urgent Caesarean section was not insignificant (12.3%), but compares favourably with national data from 2008–10 indicating that 16% of all nulliparous women planning OU birth had an intrapartum Caesarean section.[12] In a national audit in England and Wales in 2015–16, 2.6% of **all** women giving birth experienced a severe PPH, compared with 5.3% in the nulliparous severely obese women in our study.[3]

With an increasingly obese pregnant population,[3] and limited evidence about effective interventions to improve outcomes,[5–7] the findings of this study are important because they provide evidence about what is appropriate care for this group and demonstrate that selected multiparous women with BMI between 35.1-40kg/m^2^ can safely plan birth in an AMU. With clear admission criteria and careful care planning, access to AMU care provides an opportunity to reduce intervention and improve outcomes for substantial proportions of women with BMI between 35.1-40kg/m^2^. It is possible that this could also be associated with cost savings compared with planned OU birth.[27] Qualitative studies suggest that, like all women,[18] obese women want to have as normal a birth as possible[28] and that medicalisation of their pregnancy experience can leave obese women feeling negative about their carers and their experience.[29, 30] While there is relatively little evidence about obese women’s experience of care during labour and birth, providing more obese women with access to midwifery led care during labour and birth has the potential to improve their birth experience and outcomes.

### Conclusions and implications for policy and practice

Admission of severely obese women to AMUs in the UK is widespread, but there is evidence of ‘selection’, for example of women with BMI between 35.1-40kg/m^2^. Because of this apparent selection the results of our study should not be considered applicable to women with BMI>40kg/m^2^.

Our results indicate no evidence of increased risk associated with planning birth in an AMU for carefully selected multiparous women with BMI between 35.1-40kg/m^2^, and rates of vaginal birth were high. This is likely to bring significant benefits in terms of their likelihood of giving birth without intervention, compared with planning birth in an OU.

Nulliparous women with BMI between 35.1-40kg/m^2^ should be advised that they *may* have an increased risk of having an obstetric intervention or adverse maternal outcome, as defined in our study, and that, in particular, they have a potential increased risk of having a more urgent Caesarean section or a severe PPH compared with other women admitted to AMUs. The absolute risks of these adverse outcomes can also be used to inform women’s decision-making in consultation with their care providers.

## Supporting information

S1 Table Categorisation of potential confounders. (DOCX)

S2 Table Sensitivity analysis: Primary outcome in restricted population (including only those severely obese women for which comparison women were appropriately selected). (DOCX)

S3 Table Primary outcome in ‘otherwise healthy’ women. (DOCX)

S4 Table Secondary maternal outcomes in severely obese and comparison women. (DOCX)

S5 Table Reasons for transfer and reasons for intrapartum Caesarean section in severely obese and comparison women. (DOCX)

S6 Table Reasons for transfer, obstetric interventions, mode of birth and place of birth by postpartum haemorrhage ≥1500ml in nulliparous severely obese women (n = 312). (DOCX)

S7 Table Neonatal outcomes in babies of severely obese and comparison women. (DOCX)

## References

[1] Poston L, Caleyachetty R, Cnattingius S, Corvalán C, Uauy R, Herring S, et al Preconceptional and maternal obesity: epidemiology and health consequences. The Lancet Diabetes & Endocrinology. 2016;4(12):1025–36. 10.1016/S2213-8587(16)30217-0. 27743975

[2] Denison FC, Norwood P, Bhattacharya S, Duffy A, Mahmood T, Morris C, et al Association between maternal body mass index during pregnancy, short-term morbidity, and increased health service costs: a population-based study. BJOG: An International Journal of Obstetrics & Gynaecology. 2014;121(1):72–82. 10.1111/1471-0528.12443 24102880 10.1111/1471-0528.12443

[3] NMPA Project Team. National Maternity and Perinatal Audit: Clinical Report 2017. London: RCOG, 2017.

[4] Marchi J, Berg M, Dencker A, Olander EK, Begley C. Risks associated with obesity in pregnancy, for the mother and baby: a systematic review of reviews. Obesity Reviews. 2015;16(8):621–38. 10.1111/obr.12288 WOS:000358042200001. 26016557 10.1111/obr.12288

[5] Kalliala I, Markozannes G, Gunter MJ, Paraskevaidis E, Gabra H, Mitra A, et al Obesity and gynaecological and obstetric conditions: umbrella review of the literature. BMJ. 2017;359 10.1136/bmj.j4511 29074629 10.1136/bmj.j4511 PMC5656976

[6] Muktabhant B, Lawrie TA, Lumbiganon P, Laopaiboon M. Diet or exercise, or both, for preventing excessive weight gain in pregnancy. Cochrane Database of Systematic Reviews. 2015;(6). 10.1002/14651858.CD007145.pub3 CD007145. 26068707 10.1002/14651858.CD007145.pub3 PMC9428894

[7] Thangaratinam S, Rogozińska E, Jolly K, Glinkowski S, Roseboom T, Tomlinson JW, et al Effects of interventions in pregnancy on maternal weight and obstetric outcomes: meta-analysis of randomised evidence. BMJ: British Medical Journal. 2012;344 10.1136/bmj.e2088 22596383 10.1136/bmj.e2088 PMC3355191

[8] National Institute for Health and Care Excellence. Intrapartum Care: Care of Healthy Women and their Babies During Childbirth NICE guideline CG190. London: NICE, 2014.

[9] Hollowell J, Pillas D, Rowe R, Linsell L, Knight M, Brocklehurst P. The impact of maternal obesity on intrapartum outcomes in otherwise low risk women: secondary analysis of the Birthplace national prospective cohort study. BJOG: An International Journal of Obstetrics & Gynaecology. 2013; 10.1111/1471-0528.12437 24034832 10.1111/1471-0528.12437 PMC3906828

[10] Daemers DOA, Wijnen HAA, van Limbeek EBM, Budé LM, Nieuwenhuijze MJ, Spaanderman MEA, et al The impact of obesity on outcomes of midwife-led pregnancy and childbirth in a primary care population: a prospective cohort study. BJOG: An International Journal of Obstetrics & Gynaecology. 2014;121(11):1403–14. 10.1111/1471-0528.12684 24618305 10.1111/1471-0528.12684

[11] Vieira MC, White SL, Patel N, Seed PT, Briley AL, Sandall J, et al Prediction of uncomplicated pregnancies in obese women: a prospective multicentre study. BMC Medicine. 2017;15(1):194 10.1186/s12916-017-0956-8 29096631 10.1186/s12916-017-0956-8 PMC5669007

[12] Birthplace in England Collaborative Group. Perinatal and maternal outcomes by planned place of birth for healthy women with low risk pregnancies: the Birthplace in England national prospective cohort study. British Medical Journal (Clinical Research Ed). 2011;343:d7400 10.1136/bmj.d7400 22117057 10.1136/bmj.d7400 PMC3223531

[13] Rowe R. Birthplace terms and definitions: consensus process Birthplace in England research programme. Final report part 2. London: NIHR Service Delivery and Organisation programme, 2011.

[14] Walsh D, Spiby H, Grigg CP, Dodwell M, McCourt C, Culley L, et al Mapping midwifery and obstetric units in England. Midwifery. 2018;56:9–16. 10.1016/j.midw.2017.09.009 . 29024869 10.1016/j.midw.2017.09.009

[15] Rowe RE, Kurinczuk JJ, Hollowell J, Knight M. The UK Midwifery Study System (UKMidSS): a programme of work to establish a research infrastructure to carry out national studies of uncommon conditions and events in midwifery units. BMC Pregnancy and Childbirth. 2016;16(1):1–6. 10.1186/s12884-016-0868-1 27080858 10.1186/s12884-016-0868-1 PMC4832539

[16] Hollowell J, Rowe RE, Townend J, Knight M, Li Y, Linsell L, et al The Birthplace in England Research Programme: further analyses to enhance policy and service delivery decision-making for planned place of birth. Health Services and Delivery Research [Internet]. 2015; 3(36):[312 p.]. Available from: http://journalslibrary.nihr.ac.uk/hsdr/hsdr03360. 26334076

[17] National Institute for Health and Clinical Excellence. Caesarean Section NICE guideline CG132. London: NICE, 2011. 31855333

[18] Downe S, Finlayson K, Oladapo O, Bonet M, Gülmezoglu AM. What matters to women during childbirth: A systematic qualitative review. PLOS ONE. 2018;13(4):e0194906 10.1371/journal.pone.0194906 29772012 10.1371/journal.pone.0197791 PMC5957347

[19] World Health Organisation. World Health Organisation Global Database on Body Mass Index: BMI classification. Geneva: WHO; 2006 Available from: www.who.int/bmi/index.jsp?introPage=intro_3.html.

[20] Hollowell J, Puddicombe D, Rowe R, Linsell L, Hardy P, Stewart M, et al The Birthplace national prospective cohort study: perinatal and maternal outcomes by planned place of birth Birthplace in England research programme. Final report part 4. NIHR service delivery and organisation programme. London: NIHR Service Delivery and Organisation programme, 2011.

[21] Office for National Statistics. Standard Occupational Classification 2010 Volume 3 The National Statistics Socio-economic Classificiation: (rebased on the SOC2010) User Manual. Basingstoke: Palgrave Macmillan; 2010.

[22] HM Revenue & Customs. Personal tax credits: Children in low-income familes local measure: 2013 snapshot as at 31 August 2013 London: HMRC; 2015 [cited 2015]. Available from: https://www.gov.uk/government/statistics/personal-tax-credits-children-in-low-income-families-local-measure-2013-snapshot-as-at-31-august-2013.

[23] Bose P, Regan F, Paterson-Brown S. Improving the accuracy of estimated blood loss at obstetric haemorrhage using clinical reconstructions. BJOG: An International Journal of Obstetrics & Gynaecology. 2006;113(8):919–24. 10.1111/j.1471-0528.2006.01018.x 16907938 10.1111/j.1471-0528.2006.01018.x

[24] Kominiarek MA, Zhang J, VanVeldhuisen P, Troendle J, Beaver J, Hibbard JU. Contemporary labor patterns: the impact of maternal body mass index. American Journal of Obstetrics and Gynecology. 2011;205(3):244.e1–.e8. 10.1016/j.ajog.2011.06.014 21798510 10.1016/j.ajog.2011.06.014 PMC3212654

[25] Cedergren MI. Non-elective caesarean delivery due to ineffective uterine contractility or due to obstructed labour in relation to maternal body mass index. European Journal of Obstetrics Gynecology and Reproductive Biology. 2009;145(2):163–6. 10.1016/j.ejogrb.2009.05.022 19525054 10.1016/j.ejogrb.2009.05.022

[26] Ellekjaer KL, Bergholt T, Løkkegaard E. Maternal obesity and its effect on labour duration in nulliparous women: a retrospective observational cohort study. BMC Pregnancy and Childbirth. 2017;17(1):222 10.1186/s12884-017-1413-6 28701155 10.1186/s12884-017-1413-6 PMC5508690

[27] Schroeder E, Petrou S, Patel N, Hollowell J, Puddicombe D, Redshaw M, et al Cost effectiveness of alternative planned places of birth in woman at low risk of complications: evidence from the Birthplace in England national prospective cohort study. BMJ: British Medical Journal. 2012;344 10.1136/bmj.e2292 22517916 10.1136/bmj.e2292 PMC3330132

[28] Nyman VMK, Prebensen ÅK, Flensner GEM. Obese women's experiences of encounters with midwives and physicians during pregnancy and childbirth. Midwifery. 2010;26(4):424–9. 10.1016/j.midw.2008.10.008. 19100667

[29] Smith D, Lavender T. The maternity experience for women with a body mass index ≥ 30 kg/m2: a meta-synthesis. BJOG: An International Journal of Obstetrics & Gynaecology. 2011;118(7):779–89. 10.1111/j.1471-0528.2011.02924.x. 21385305 10.1111/j.1471-0528.2011.02924.x

[30] Furber CM, McGowan L. A qualitative study of the experiences of women who are obese and pregnant in the UK. Midwifery. 27(4):437–44. 10.1016/j.midw.2010.04.001 20483513 10.1016/j.midw.2010.04.001

