# Supplementary material for: Outcomes for women with BMI>35kg/m2 admitted for labour care to alongside midwifery units in the UK: A national prospective cohort study using the UK Midwifery Study System (UKMidSS)
Source: PLoS One. 2018 Dec 4;13(12):e0208041. doi: 10.1371/journal.pone.0208041 (PMC6279017; doi:10.1371/journal.pone.0208041)
Supplement: S1 Table — (DOCX) [file pone.0208041.s001.docx]

*S1 Table: Categorisation of potential confounders*

| **Covariate** | **Response categories** | | **Notes** |
| --- | --- | --- | --- |
| Maternal age (years) | 1 | <25 | Because of small numbers for some secondary outcomes^a^ recoded as: <25, 25-29, >29; or^b^ recoded as: <30, ≥30. |
|  | 2 | 25-29 |  |
|  | 3 | 30-34 |  |
|  | 4 | >34 |  |
| Ethnic group | 1 | White | Because of small numbers for some secondary outcomes^c^ recoded as: White, Other, Not recorded; or^d^ recoded as: White, Other, with Not recorded as missing; or^e^ White, Asian, Black, Other, with Not recorded recoded as missing. |
|  | 2 | Asian |  |
|  | 3 | Black |  |
|  | 4 | Other |  |
|  | 5 | Not recorded |  |
| Gestational age at | 2 | 35-38 |  |
| admission (completed | 3 | 39 |  |
| weeks) | 1 | 40 |  |
|  | 4 | 41-42 |  |
| Children in Low-income | 1 | 1^st^ (least deprived) |  |
| Families Measure quintile | 2 | 2^nd^ |  |
|  | 3 | 3^rd^ |  |
|  | 4 | 4^th^ |  |
|  | 5 | 5^th^ (most deprived) |  |
| Previous pregnancies ≥ | 1 | 0 Nulliparous | Categories 1-4 used in pooled analyses combining nulliparous and parous women.  Only categories 2-4 used in analyses of parous women.  Because of small numbers for some secondary outcomes^f^ recoded as: 1 previous, ≥2 previous; or^g^ recoded as: Nulliparous, 1 previous, ≥2previous. |
| 24 weeks | 2 | 1 previous |  |
|  | 3 | 2 previous |  |
|  | 4 | ≥3 previous |  |
| Pre-existing risk factors | 1 | None | Because of small numbers for some secondary outcomes^h^ recoded as: None, ≥1. |
|  | 2 | ≥1 clear |  |
|  | 3 | ≥1 possible |  |

^a^ Shoulder dystocia (nulliparous); general anaesthesia (nulliparous)

^b^ Apgar score (multiparous)

^c^ Shoulder dystocia (nulliparous & overall); general anaesthesia (nulliparous); instrumental delivery (nulliparous & overall); intrapartum caesarean (multiparous); category 1 or 2 caesarean (multiparous); PPH (nulliparous); maternal blood transfusion (nulliparous, multiparous & overall); maternal admission (nulliparous); Apgar score (multiparous)

^d^ Shoulder dystocia (nulliparous); instrumental delivery (multiparous); neonatal unit admission (nulliparous)

^e^ Neonatal unit admission (multiparous & overall)

^f^ General anaesthesia (multiparous); Apgar score (multiparous)

^g^ General anaesthesia (overall); Apgar score (overall)

^h^ Shoulder dystocia (nulliparous); augmentation (multiparous); Apgar score (nulliparous)
