# Supplementary material for: Outcomes for women with BMI>35kg/m2 admitted for labour care to alongside midwifery units in the UK: A national prospective cohort study using the UK Midwifery Study System (UKMidSS)
Source: PLoS One. 2018 Dec 4;13(12):e0208041. doi: 10.1371/journal.pone.0208041 (PMC6279017; doi:10.1371/journal.pone.0208041)
Supplement: S2 Table — (DOCX) [file pone.0208041.s002.docx]

*S2 Table: Sensitivity analysis: Primary outcome in restricted population (including only those ‘severely obese’ women for which comparison women were appropriately selected)*

|  | Events | Births |  | | Unadjusted | | Adjusted^a^ | |
| --- | --- | --- | --- | --- | --- | --- | --- | --- |
|  | n | n | % | (95% CI) | RR | (95% CI) | RR | (95% CI) |
| **Adverse maternal outcome composite^b^** |  |  |  |  |  |  |  |  |
| Overall |  |  |  |  |  |  |  |  |
| Comparison group | 394 | 1945 | 20.3 | (18.5-22.0) | 1 |  | 1 |  |
| Severely obese women | 151 | 983 | 15.4 | (13.1-17.6) | 0.76 | (0.62-0.93) | 1.02 | (0.85-1.22) |
| Wald test for interaction |  |  |  |  |  |  |  | p=0.09^c^ |
| Nulliparous |  |  |  |  |  |  |  |  |
| Comparison group | 309 | 889 | 34.8 | (31.6-37.9) | 1 |  |  |  |
| Severely obese women | 106 | 279 | 38.0 | (32.3-43.7) | 1.09 | (0.94-1.27) | 1.14 | (0.97-1.34) |
| Multiparous |  |  |  |  |  |  |  |  |
| Comparison group | 85 | 1053 | 8.1 | (6.4-9.7) | 1 |  | 1 |  |
| Severely obese women | 44 | 703 | 6.3 | (4.5-8.1) | 0.78 | (0.51-1.19) | 0.76 | (0.49-1.18) |

^a^ Adjusted for maternal age, ethnic group, Children in Low Income Families Measure quintile, gestation at admission, risk status and parity where appropriate

^b^ Comprising: augmentation, instrumental birth, Caesarean, maternal blood transfusion, 3^rd^/4^th^ degree tear, maternal admission to higher level care

^c^ *p* value for interaction, adjusted for maternal age, ethnic group, Children in Low Income Families Measure quintile, gestation at admission, risk status and parity (binary)
