# Supplementary material for: Outcomes for women with BMI>35kg/m2 admitted for labour care to alongside midwifery units in the UK: A national prospective cohort study using the UK Midwifery Study System (UKMidSS)
Source: PLoS One. 2018 Dec 4;13(12):e0208041. doi: 10.1371/journal.pone.0208041 (PMC6279017; doi:10.1371/journal.pone.0208041)
Supplement: S3 Table — (DOCX) [file pone.0208041.s003.docx]

*S3 Table: Primary outcome in ‘otherwise healthy’ women^a^*

|  | Events | Births |  | | Unadjusted | | Adjusted^b^ | |
| --- | --- | --- | --- | --- | --- | --- | --- | --- |
|  | n | n | % | (95% CI) | RR | (95% CI) | RR | (95% CI) |
| **Adverse maternal outcome composite^c^** |  |  |  |  |  |  |  |  |
| Overall |  |  |  |  |  |  |  |  |
| Comparison group | 365 | 1794 | 20.4 | (18.5-22.2) | 1 |  | 1 |  |
| Severely obese women | 144 | 974 | 14.8 | (12.6-17.0) | 0.73 | (0.59-0.90) | 0.99 | (0.82-1.20) |
| Wald test for interaction |  |  |  |  |  |  |  | p=0.05^d^ |
| Nulliparous |  |  |  |  |  |  |  |  |
| Comparison group | 292 | 844 | 34.6 | (31.4-37.8) | 1 |  |  |  |
| Severely obese women | 107 | 285 | 37.5 | (31.9-43.2) | 1.09 | (0.93-1.27) | 1.13 | (0.96-1.34) |
| Multiparous |  |  |  |  |  |  |  |  |
| Comparison group | 73 | 948 | 7.7 | (6.0-9.4) | 1 |  | 1 |  |
| Severely obese women | 35 | 687 | 5.1 | (3.4-6.7) | 0.66 | (0.41-1.08) | 0.67 | (0.40-1.11) |

^a^ Restricted to the sub-group of women not identified as having any pre-existing risk factors (apart from BMI>35kg/m^2^ in severely obese)

^b^ Adjusted for maternal age, ethnic group, Children in Low Income Families Measure quintile, gestation at admission, and parity where appropriate

^c^ Comprising: augmentation, instrumental birth, Caesarean, maternal blood transfusion, 3^rd^/4^th^ degree tear, maternal admission to higher level care

^d^ *p* value for interaction, adjusted for maternal age, ethnic group, Children in Low Income Families Measure quintile, gestation at admission, and parity (binary)
