# Supplementary material for: Outcomes for women with BMI>35kg/m2 admitted for labour care to alongside midwifery units in the UK: A national prospective cohort study using the UK Midwifery Study System (UKMidSS)
Source: PLoS One. 2018 Dec 4;13(12):e0208041. doi: 10.1371/journal.pone.0208041 (PMC6279017; doi:10.1371/journal.pone.0208041)
Supplement: S4 Table — (DOCX) [file pone.0208041.s004.docx]

*S4 Table: Secondary maternal outcomes in ‘severely obese’ and comparison women*

|  | Events | Births |  | | Unadjusted | | Adjusted^a^ | |
| --- | --- | --- | --- | --- | --- | --- | --- | --- |
|  | n | n | % | (95% CI) | RR | (99% CI) | RR | (99% CI) |
| **Transfer (during labour or after birth)** |  |  |  |  |  |  |  |  |
| Overall |  |  |  |  |  |  |  |  |
| Comparison group | 509 | 1949 | 26.1 | (24.2-28.1) | 1 |  | 1 |  |
| Severely obese women | 270 | 1122 | 24.1 | (21.6-26.6) | 0.92 | (0.74-1.15) | 1.15 | (0.96-1.37) |
| Wald test for interaction |  |  |  |  |  |  |  | p=0.85^b^ |
| Nulliparous |  |  |  |  |  |  |  |  |
| Comparison group | 375 | 890 | 42.1 | (38.9-45.4) | 1 |  | 1 |  |
| Severely obese women | 151 | 312 | 48.4 | (42.8-54.0) | 1.15 | (0.96-1.37) | 1.18 | (0.98-1.43) |
| Multiparous |  |  |  |  |  |  |  |  |
| Comparison group | 134 | 1056 | 12.7 | (10.7-14.7) | 1 |  | 1 |  |
| Severely obese women | 118 | 808 | 14.6 | (12.2-17.0) | 1.15 | (0.84-1.57) | 1.12 | (0.84-1.49) |
| **Shoulder dystocia** |  |  |  |  |  |  |  |  |
| Overall |  |  |  |  |  |  |  |  |
| Comparison group | 28 | 1949 | 1.4 | (0.9-2.0) | 1 |  | 1 |  |
| Severely obese women | 15 | 1122 | 1.3 | (0.7-2.0) | 0.93 | (0.39-2.22) | 0.82 | (0.33-2.03) |
| Wald test for interaction |  |  |  |  |  |  |  | p=0.88^b^ |
| Nulliparous |  |  |  |  |  |  |  |  |
| Comparison group | 11 | 890 | 1.2 | (0.51-2.0) | 1 |  |  |  |
| Severely obese women | 3 | 312 | 1.0 | (0.0-2.1) | 0.78 | (0.13-4.52) | 0.79 | (0.14-4.51) |
| Multiparous |  |  |  |  |  |  |  |  |
| Comparison group | 17 | 1056 | 1.6 | (0.85-2.4) | 1 |  | 1 |  |
| Severely obese women | 12 | 808 | 1.5 | (0.65-2.3) | 0.92 | (0.36-2.34) | 0.84 | (0.31-2.23) |
| **Augmentation with syntocinon** |  |  |  |  |  |  |  |  |
| Overall |  |  |  |  |  |  |  |  |
| Comparison group | 157 | 1946 | 8.1 | (6.9-9.3) | 1 |  | 1 |  |
| Severely obese women | 80 | 1121 | 7.1 | (5.6-8.6) | 0.88 | (0.57-1.37) | 1.28 | (0.88-1.86) |
| Wald test for interaction |  |  |  |  |  |  |  | p=0.79^b^ |
| Nulliparous |  |  |  |  |  |  |  |  |
| Comparison group | 143 | 888 | 16.1 | (13.7-18.5) | 1 |  | 1 |  |
| Severely obese women | 64 | 311 | 20.6 | (16.1-25.1) | 1.28 | (0.87-1.88) | 1.29 | (0.88-1.88) |
| Multiparous |  |  |  |  |  |  |  |  |
| Comparison group | 14 | 1055 | 1.3 | (0.64-2.0) | 1 |  | 1 |  |
| Severely obese women | 16 | 808 | 2.0 | (1.0-2.9) | 1.49 | (0.61-3.63) | 1.28 | (0.42-3.90) |
| **General anaesthesia** |  |  |  |  |  |  |  |  |
| Overall |  |  |  |  |  |  |  |  |
| Comparison group | 14 | 1946 | 0.72 | (0.34-1.1) | 1 |  | 1 |  |
| Severely obese women | 6 | 1121 | 0.54 | (0.11-0.96) | 0.74 | (0.20-2.84) | 0.92 | (0.24-3.55) |
| Wald test for interaction |  |  |  |  |  |  |  | p=0.57^b^ |
| Nulliparous |  |  |  |  |  |  |  |  |
| Comparison group | 10 | 888 | 1.1 | (0.4-1.8) | 1 |  | 1 |  |
| Severely obese women | 4 | 311 | 1.3 | (0.03-2.5) | 1.14 | (0.25-5.15) | 1.08 | (0.36-3.21) |
| Multiparous |  |  |  |  |  |  |  |  |
| Comparison group | 4 | 1055 | 0.38 | (0-0.75) | 1 |  | 1 |  |
| Severely obese women | 2 | 808 | 0.25 | (0-0.60) | 0.65 | (0.04-11.0) | 0.68^c^ | (0.09-4.86) |
| **Vaginal birth** |  |  |  |  |  |  |  |  |
| Overall |  |  |  |  |  |  |  |  |
| Comparison group | 1688 | 1949 | 86.6 | (85.1-88.1) | 1 |  | 1 |  |
| Severely obese women | 1014 | 1122 | 90.4 | (88.6-92.1) | 1.04 | (1.01-1.08) | 0.99 | (0.96-1.03) |
| Wald test for interaction |  |  |  |  |  |  |  | p=0.31^b^ |
| Nulliparous |  |  |  |  |  |  |  |  |
| Comparison group | 662 | 890 | 74.4 | (71.5-77.3) | 1 |  | 1 |  |
| Severely obese women | 226 | 312 | 72.4 | (67.5-77.4) | 0.97 | (0.89-1.07) | 0.97 | (0.88-1.07) |
| Multiparous |  |  |  |  |  |  |  |  |
| Comparison group | 1023 | 1056 | 96.9 | (95.8-97.9) | 1 |  | 1 |  |
| Severely obese women | 786 | 808 | 97.3 | (96.2-98.4) | 1.00 | (0.98-1.03) | 1.00 | (0.98-1.03) |
| **Straightforward vaginal birth** |  |  |  |  |  |  |  |  |
| Overall |  |  |  |  |  |  |  |  |
| Comparison group | 1610 | 1948 | 82.7 | (81.0-84.3) | 1 |  | 1 |  |
| Severely obese women | 988 | 1119 | 88.3 | (86.4-90.2) | 1.07 | (1.02-1.11) | 1.01 | (0.97-1.05) |
| Wald test for interaction |  |  |  |  |  |  |  | p=0.11^b^ |
| Nulliparous |  |  |  |  |  |  |  |  |
| Comparison group | 621 | 890 | 69.8 | (66.8-72.8) | 1 |  | 1 |  |
| Severely obese women | 212 | 312 | 67.9 | (62.7-73.2) | 0.97 | (0.89-1.07) | 0.96 | (0.86-1.06) |
| Multiparous |  |  |  |  |  |  |  |  |
| Comparison group | 986 | 1055 | 93.5 | (92.0-95.0) | 1 |  | 1 |  |
| Severely obese women | 776 | 806 | 96.3 | (95.0-97.6) | 1.03 | (0.99-1.07) | 1.03 | (0.99-1.07) |
| **Instrumental birth** |  |  |  |  |  |  |  |  |
| Overall |  |  |  |  |  |  |  |  |
| Comparison group | 181 | 1949 | 9.3 | (8.0-10.6) | 1 |  | 1 |  |
| Severely obese women | 55 | 1122 | 4.9 | (3.6-6.2) | 0.53 | (0.35-0.80) | 0.79 | (0.53-1.19) |
| Wald test for interaction |  |  |  |  |  |  |  | p=0.31^b^ |
| Nulliparous |  |  |  |  |  |  |  |  |
| Comparison group | 155 | 890 | 17.4 | (14.9-19.9) | 1 |  | 1 |  |
| Severely obese women | 43 | 312 | 13.8 | (9.9-17.6) | 0.79 | (0.51-1.22) | 0.83 | (0.53-1.30) |
| Multiparous |  |  |  |  |  |  |  |  |
| Comparison group | 26 | 1056 | 2.5 | (1.5-3.4) | 1 |  | 1 |  |
| Severely obese women | 12 | 808 | 1.5 | (0.6-2.3) | 0.60 | (0.24-1.49) | 0.60 | (0.22-1.61) |
| **Third/fourth degree perineal trauma** |  |  |  |  |  |  |  |  |
| Wald test for interaction |  |  |  |  |  |  |  | p=0.02^b^ |
| Nulliparous |  |  |  |  |  |  |  |  |
| Comparison group | 52 | 890 | 5.8 | (4.3-7.4) | 1 |  | 1 |  |
| Severely obese women | 19 | 312 | 6.1 | (3.4-8.8) | 1.04 | (0.56-1.93) | 1.24 | (0.68-2.26) |
| Multiparous |  |  |  |  |  |  |  |  |
| Comparison group | 28 | 1055 | 2.7 | (1.7-3.6) | 1 |  | 1 |  |
| Severely obese women | 6 | 806 | 0.7 | (0.15-1.3) | 0.28 | (0.07-1.09) | 0.32 | (0.08-1.26) |
| **Maternal blood transfusion** |  |  |  |  |  |  |  |  |
| Overall |  |  |  |  |  |  |  |  |
| Comparison group | 18 | 1948 | 0.9 | (0.5-1.3) | 1 |  | 1 |  |
| Severely obese women | 12 | 1119 | 1.1 | (0.5-1.7) | 1.16 | (0.47-2.85) | 1.32 | (0.54-3.18) |
| Wald test for interaction |  |  |  |  |  |  |  | p=0.07^b^ |
| Nulliparous |  |  |  |  |  |  |  |  |
| Comparison group | 8 | 890 | 0.9 | (0.3-1.5) | 1 |  | 1 |  |
| Severely obese women | 8 | 312 | 2.6 | (0.8-4.3) | 2.85 | (0.81-10.10) | 2.88 | (0.77-10.80) |
| Multiparous |  |  |  |  |  |  |  |  |
| Comparison group | 10 | 1055 | 1.0 | (0.4-1.5) | 1 |  | 1 |  |
| Severely obese women | 4 | 806 | 0.5 | (0.01-1.0) | 0.52 | (0.09-2.96) | 0.50 | (0.11-2.17) |
| **Maternal admission for higher level care** |  |  |  |  |  |  |  |  |
| Overall |  |  |  |  |  |  |  |  |
| Comparison group | 37 | 1947 | 1.9 | (1.3, 2.5) | 1 |  | 1 |  |
| Severely obese women | 21 | 1120 | 1.9 | (1.1, 2.7) | 0.99 | (0.40, 2.45) | 0.98 | (0.43- 2.26) |
| Wald test for interaction |  |  |  |  |  |  |  | p=0.14^b^ |
| Nulliparous |  |  |  |  |  |  |  |  |
| Comparison group | 20 | 890 | 2.3 | (1.3, 3.2) | 1 |  | 1 |  |
| Severely obese women | 10 | 312 | 3.2 | (1.2, 5.2) | 1.43 | (0.44, 4.58) | 1.34 | (0.44, 4.11) |
| Multiparous |  |  |  |  |  |  |  |  |
| Comparison group | 17 | 1054 | 1.6 | (0.9, 2.4) | 1 |  | 1 |  |
| Severely obese women | 9 | 806 | 1.1 | (0.4, 1.8) | 0.69 | (0.24, 1.97) | 0.71 | (0.27-1.86) |

^a^ Adjusted for maternal age, ethnic group, Children in Low Income Families Measure quintile, gestation at admission, risk status, and parity where appropriate

^b^ *p* value for interaction, adjusted for maternal age, ethnic group, Children in Low Income Families Measure quintile, gestation at admission, risk status and parity (binary)

^c^ Adjusted for maternal age, gestation at admission, risk status, and parity only because of small numbers
