# Supplementary material for: Outcomes for women with BMI>35kg/m2 admitted for labour care to alongside midwifery units in the UK: A national prospective cohort study using the UK Midwifery Study System (UKMidSS)
Source: PLoS One. 2018 Dec 4;13(12):e0208041. doi: 10.1371/journal.pone.0208041 (PMC6279017; doi:10.1371/journal.pone.0208041)
Supplement: S5 Table — (DOCX) [file pone.0208041.s005.docx]

**S5 Table. Reasons for transfer and reasons for intrapartum Caesarean section in severely obese and comparison women**

|  | **Severely obese women** | | **Comparison group** | |  |
| --- | --- | --- | --- | --- | --- |
|  | n | % | n | % | p value^a^ |
| **Primary reason for transfer (n=774)** | |  |  |  | 0.04 |
| Maternal concerns during labour^b^ | 48 | 18.0 | 62 | 12.2 |  |
| Fetal concerns^c^ | 80 | 30.0 | 125 | 24.7 |  |
| Slow progress of labour | 64 | 24.0 | 145 | 28.6 |  |
| Epidural/pain relief | 30 | 11.2 | 79 | 15.6 |  |
| Maternal concerns postpartum^d^ | 45 | 16.8 | 96 | 18.9 |  |
| Missing | 2 |  | 3 |  |  |
| **Primary reason for Caesarean section (n=133)** | |  |  |  | 0.19 |
| Fetal compromise | 27 | 50.9 | 31 | 38.8 |  |
| Slow progress of labour | 21 | 39.6 | 33 | 41.3 |  |
| Other reason^f^ | 5 | 9.4 | 16 | 20.0 |  |
| Missing | 0 |  | 0 |  |  |

^a^ Χ^2^ test

^b^ e.g. hypertension, prolonged rupture of membranes, pyrexia

^c^ e.g. fetal heart rate abnormalities, meconium

^d^ e.g. retained placenta, PPH, perineal trauma repair

^f^ e.g. abnormal presentation, maternal compromise, other
