# Supplementary material for: Outcomes for women with BMI>35kg/m2 admitted for labour care to alongside midwifery units in the UK: A national prospective cohort study using the UK Midwifery Study System (UKMidSS)
Source: PLoS One. 2018 Dec 4;13(12):e0208041. doi: 10.1371/journal.pone.0208041 (PMC6279017; doi:10.1371/journal.pone.0208041)
Supplement: S6 Table — (DOCX) [file pone.0208041.s006.docx]

**S6 Table: Reasons for transfer, obstetric interventions, mode of birth and place of birth by postpartum haemorrhage ≥1500ml in nulliparous severely obese women (n=312)**

|  | **Postpartum haemorrhage ≥1500ml** | | | |  |
| --- | --- | --- | --- | --- | --- |
|  | **No** | | **Yes** | |  |
|  | n | % | n | % | p value^a^ |
| **Reason for transfer^b^** |  |  |  |  | <0.001 |
| Not transferred | 163 | 55.1 | 0 | 0 |  |
| Delay in labour | 34 | 11.5 | 8 | 50.0 |  |
| Other reason | 99 | 33.5 | 8 | 50.0 |  |
| **Augmentation with syntocinon** |  |  |  |  | <0.001 |
| No | 240 | 81.4 | 7 | 43.8 |  |
| Yes | 55 | 18.6 | 9 | 56.3 |  |
| **Epidural** |  |  |  |  | <0.001 |
| No | 214 | 72.5 | 3 | 18.8 |  |
| Yes | 81 | 27.5 | 13 | 81.3 |  |
| **Mode of birth** |  |  |  |  | 0.001 |
| Spontaneous vaginal | 221 | 74.7 | 5 | 31.3 |  |
| Instrumental | 37 | 12.5 | 6 | 37.5 |  |
| Caesarean | 38 | 12.8 | 5 | 31.3 |  |
| **Place of birth** |  |  |  |  | 0.001 |
| Alongside Midwifery Unit | 80 | 60.8 | 3 | 18.8 |  |
| Obstetric Unit | 116 | 39.2 | 13 | 81.3 |  |

^a^ Χ^2^ test

^b^ Transfer during labour or within 24 hours of birth. Reasons: Delay in labour (confirmed delay in 1^st^/2^nd^ stage); Other reason (hypertension; significant meconium; epidural/pain relief; fetal heart rate abnormalities in 1^st^/2^nd^ stage; retained placenta; perineal trauma repair; other).
