# Supplementary material for: Outcomes for women with BMI>35kg/m2 admitted for labour care to alongside midwifery units in the UK: A national prospective cohort study using the UK Midwifery Study System (UKMidSS)
Source: PLoS One. 2018 Dec 4;13(12):e0208041. doi: 10.1371/journal.pone.0208041 (PMC6279017; doi:10.1371/journal.pone.0208041)
Supplement: S7 Table — (DOCX) [file pone.0208041.s007.docx]

**S7 Table. Neonatal outcomes in babies of severely obese and comparison women**

|  | Events | Births |  | | Unadjusted | | Adjusted^a^ | |
| --- | --- | --- | --- | --- | --- | --- | --- | --- |
|  | n | n | % | (95% CI) | RR | (99% CI) | RR | (99% CI) |
| **Apgar <7 at 5 minutes** |  |  |  |  |  |  |  |  |
| Overall |  |  |  |  |  |  |  |  |
| Comparison group | 18 | 1939 | 0.9 | (0.5, 1.4) | 1 |  | 1 |  |
| Severely obese women | 10 | 1120 | 0.9 | (0.3, 1.4) | 0.96 | (0.41, 2.26) | 1.08 | (0.40, 2.91) |
| Wald test for interaction |  |  |  |  |  |  |  | p=0.73^b^ |
| Nulliparous |  |  |  |  |  |  |  |  |
| Comparison group | 13 | 882 | 1.5 | (0.7, 2.3) | 1 |  | 1 |  |
| Severely obese women | 6 | 312 | 1.9 | (0.4, 3.5) | 1.30 | (0.44, 3.85) | 1.33^c^ | (0.47, 3.72) |
| Multiparous |  |  |  |  |  |  |  |  |
| Comparison group | 5 | 1054 | 0.5 | (0.06, 0.9) | 1 |  | 1 |  |
| Severely obese women | 4 | 806 | 0.5 | (0.01, 1.0) | 1.05 | (0.15, 7.18) | 0.86 | (0.11, 7.05) |
| **Initiation of breastfeeding** |  |  |  |  |  |  |  |  |
| Overall |  |  |  |  |  |  |  |  |
| Comparison group | 1442 | 1943 | 74.2 | (72.3, 76.2) | 1 |  | 1 |  |
| Severely obese women | 732 | 1120 | 65.4 | (62.6, 68.1) | 0.88 | (0.82, 0.95) | 0.96 | (0.89, 1.03) |
| Wald test for interaction |  |  |  |  |  |  |  | p=0.35^b^ |
| Nulliparous |  |  |  |  |  |  |  |  |
| Comparison group | 693 | 886 | 78.2 | (75.5, 80.9) | 1 |  | 1 |  |
| Severely obese women | 229 | 312 | 73.4 | (68.5, 78.3) | 0.94 | (0.85, 1.04) | 0.97 | (0.87, 1.07) |
| Multiparous |  |  |  |  |  |  |  |  |
| Comparison group | 747 | 1054 | 70.9 | (68.1, 73.6) | 1 |  | 1 |  |
| Severely obese women | 502 | 806 | 62.3 | (58.9, 65.6) | 0.88 | (0.81, 0.95) | 0.92 | (0.85, 1.00) |
| **Neonatal unit admission** |  |  |  |  |  |  |  |  |
| Overall |  |  |  |  |  |  |  |  |
| Comparison group | 49 | 1943 | 2.5 | (1.8, 3.2) | 1 |  | 1 |  |
| Severely obese women | 32 | 1120 | 2.9 | (1.9, 3.8) | 1.13 | (0.58, 2.20) | 1.03 | (0.53, 1.99) |
| Wald test for interaction |  |  |  |  |  |  |  | p=0.77^b^ |
| Nulliparous |  |  |  |  |  |  |  |  |
| Comparison group | 29 | 886 | 3.3 | (2.1, 4.4) | 1 |  | 1 |  |
| Severely obese women | 12 | 312 | 3.9 | (1.7, 6.0) | 1.18 | (0.43, 3.21) | 0.92 | (0.38, 2.23) |
| Multiparous |  |  |  |  |  |  |  |  |
| Comparison group | 20 | 1054 | 1.9 | (1.1, 2.7) | 1 |  | 1 |  |
| Severely obese women | 19 | 806 | 2.4 | (1.3, 3.4) | 1.24 | (0.53, 2.90) | 1.10 | (0.46, 2.68) |

^a^ Adjusted for maternal age, ethnic group, Children in Low Income Families Measure quintile, gestation at admission, risk status, and parity where appropriate

^b^ *p* value for interaction, adjusted for maternal age, ethnic group, Children in Low Income Families Measure quintile, gestation at admission, risk status and parity (binary)

^c^ Adjusted for maternal age, Children in Low Income Families Measure quintile, gestation at admission and risk status only because of small numbers
